# Long non-coding RNA LINC01268 promotes cell growth and inhibits cell apoptosis by modulating miR-217/SOS1 axis in acute myeloid leukemia

**DOI:** 10.1590/1414-431X20209299

**Published:** 2020-06-26

**Authors:** Beili Chen, Yuchuan Li, Yuwei Nie, Ailin Tang, Qin Zhou

**Affiliations:** 1 Department of Hematology, Affiliated Hospital of Guilin Medical University, Guilin, Guangxi, China; 2 Department of Gynecology, Affiliated Hospital of Guilin Medical University, Guilin, Guangxi, China

**Keywords:** LINC01268, miR-217, SOS1, Acute myeloid leukemia, ceRNA, lncRNA

## Abstract

The aim of this study was to evaluate the pathogenic role of newly identified long non-coding (lnc)-RNA LINCO1268 in acute myeloid leukemia (AML), and investigate its therapeutic potential. The expression level of LINC01268 in AML was measured by quantitative PCR (qPCR). The viability, cell cycle progression, and apoptosis of AML cells were measured by CCK-8 assay and flow cytometry, respectively. The interaction between LINC01268 and miR-217 were predicted by the miRDB website, and then verified by luciferase reporter assay and RNA immunoprecipitation (RIP) assay. The relationship between miR-217 and SOS1 was predicted by TargetScan website, and verified by luciferase reporter assay. LINC01268 was significantly upregulated by 1.6 fold in bone marrow samples of AML patients, which was associated with poor prognosis. LINC01268 was also significantly upregulated in AML cells. LINC01268 knockdown inhibited viability and cell cycle progression but promoted apoptosis of AML cells. Furthermore, LINC01268 functioned as a ceRNA via competitively binding to miR-217, and SOS1 was identified as a target of miR-217. Moreover, LINC01268 positively regulated SOS1 expression to promote AML cell viability and cell cycle progression but inhibited apoptosis via sponging miR-217. LINC01268 promoted cell growth and inhibited cell apoptosis through modulating miR-217/SOS1 axis in AML. This study offers a novel molecular mechanism for a better understanding of the pathology of AML. LINC01268 could be considered as a potential biomarker for the therapy and diagnosis of AML.

## Introduction

Acute myeloid leukemia (AML), as a highly heterogeneous disorder, is the most common acute leukemia in adults (1). The general feature of AML is aberrant proliferation and differentiation of hematopoietic progenitor cells in the bone marrow, peripheral blood, or other tissues (2,3). For more than four decades, the standard therapy of AML includes chemotherapy (e.g., the combination of an anthracycline and cytarabine) and allogeneic stem cell transplantation (4,5). Despite the improvements in the outcomes for younger AML patients, the prognosis of the older AML patients remains poor (6). The five-year overall survival is below 50% for AML patients under the age of 60, and less than 20% for older patients (5). Therefore, new therapies are urgently needed to improve the outcome, and particularly, targeted therapies have attracted attention.

Long non-coding RNAs (lncRNAs) are defined as non-protein coding transcripts larger than 200 nucleotides (7). Accumulated evidence has suggested that lncRNAs play important roles in the regulation of gene expression (8). lncRNAs have been found to be differentially expressed in human cancers, which significantly affects cancer initiation, development, and progression (9). For instance, lncRNA HOTAIRM1 has been shown to regulate myeloid differentiation and maturation in AML through affecting integrin genes [ITGA4(CD49d) and ITGAX(CD11c)] (10). Overexpression of lncRNA PVT1 can promote apoptosis and necrosis of AML cell lines by down-regulation of c-MYC(11). lncRNA ANRIL could modulate the AdipoR1/AMPK/SIRT1 signaling pathway to regulate the development of AML (12). Thus, it seems that lncRNAs are potential biomarkers for targeted therapy of AML (13).

Long intergenic non-protein coding RNA 1268 (LINC01268), also known as LOC285758, is a newly identified lncRNA (14). LINC01268 has been found to be methylation-dependent and associated with the malignancy grade of glioma (14). A recent report has shown that LINC01268 is associated with the progression of AML (15). However, its role in the development of AML is not well known. Therefore, the effect of LINC01268 on AML and the underlying mechanism were explored in this study through a series of biological experiments.

## Material and Methods

### Human samples

The diagnosis of AML was confirmed based on morphological, cytochemical, immunophenotyping, and cytogenetic analysis of bone marrow samples, and AML was classified according to the FAB (French-American-British) criteria (16). Before the experiment, we asked for healthy volunteers to donate bone marrow for research purposes. The healthy donor had to meet the following requirements: be in good general health (no history of heart disease, asthma, diabetes, or chronic medical problems); be 18 to 60 years of age; not be pregnant; weigh less than 225 lbs; and not use any prescription drugs. A total of 98 bone marrow samples were collected from 50 untreated AML patients (15 to 88 years, excluding M3 and M7 AML subtypes) and 48 healthy donors at the Affiliated Hospital of Guilin Medical University. AML patients were followed up for 60 months after surgery. The clinical characteristics of patients are listed in Table 1. Written informed consents were obtained from all patients. The present study was approved by the Institutional Ethics Committee of Affiliated Hospital of Guilin Medical University.

**Table 1:** Correlation between LINC01268 expression and clinical characteristics of acute myeloid leukemia patients.

Clinicopathological variables	High LINC01268	Low LINC01268	P-value
Age (years)	55.13±21.76	52.19±23.37	0.945
Gender (male/female)	12/11	13/14	0.777
White blood cells (×10^9^/L)	17.72±8.67	11.68±8.80	0.001
Hemoglobin (g/L)	60.01±28.98	88.96±30.70	0.007
Platelet count (×10^9^/L)	37.7±25.18	55.83±31.69	0.001
FAB classification			0.74
M1/M2	11	10	
M4/M5	9	13	
M0/M6	3	4	
Complete remission			0.073
Yes	4	11	
No	19	16	

Data are reported as means±SD or number (chi-squared test). FAB: French, American, British criteria.

### Cell culture and cell transfection

Human bone marrow stromal cell line HS-5 and human AML cell lines (HL-60, KG-1, and Kasumi-1) were obtained from American Type Culture Collection (ATCC, USA). Cells were incubated in RPMI 1640 medium (Gibco, USA) containing 100 U/mL penicillin, 0.1 mg/mL streptomycin, and 10% fetal bovine serum (FBS, Gibco) at 37°C in a humidified incubator with 5% CO_2_.

Two different kinds of siRNAs for LINC01268 (si-LINC01268 #1 and si-LINC01268 #2) and control siRNA (si-NC) were purchased from GenePharma (China). miR-217 mimic, miRNA scrambled control (control mimic), miR-217 inhibitor, and inhibitor control (NC inhibitor) were synthesized by RiboBio (China). After growing to 80% confluence, cells were transfected with the above compounds using Lipofectamine 3000 reagent (Invitrogen, USA) following the manufacturer's instructions.

### Quantitative real-time PCR (qRT-PCR)

Total RNA from tissues and cells were isolated using Trizol reagent (Invitrogen), and reversely transcribed to cDNA with M-MLV reverse transcriptase (Invitrogen). Next, qRT-PCR was carried out using SYBR Green Mixture RT-PCR kits (Takara, China) on the ABI 7500 Fast Real-Time PCR system. Relative mRNA expression was calculated by the 2^−ΔΔCt^ method and was normalized to the internal control (β-actin). The primers are shown in Table 2.

**Table 2:** Primers for quantitative real-time PCR.

Name	Sequence (5′-3′)
LINC01268	Forward: TTGTTTTTTGAAAGTTTTTTGA
	Reverse: AAACACAAAAAACCTAACAAAAA
miR-217	Forward: TACTGCATCAGGAACTGACTGGA
	Reverse: TACTGCATCAGGAACT
SOS1	Forward: TCCACGAAGACGACCAGAAT
	Reverse: GGGGACTGTCCAAATGCTTA

### CCK-8 assay

Cell viability was determined by CCK-8 assay. Cells were separately seeded onto 96-well plates at a density of 1×10^4^ cells per well and incubated in RPMI 1640 medium (Gibco) with 10% FBS for 0, 24, 48, and 72 h. Next, CCK8 solution (10 μL) was added into each well and incubated for 4 h at 37°C. Subsequently, the sample's absorbance was detected at 450 nm by a microplate reader (EL 800 Universal Microplate reader, BioTek, USA).

### Flow cytometry analysis

For cell cycle analysis, the transfected cells were harvested, re-suspended in the binding buffer, stained with propidium iodide (PI, Sigma-Aldrich, USA ) solution, and analyzed by a FACSCalibur flow cytometer (BD Biosciences, USA).

For cell apoptosis analysis, the harvested cells were re-suspended in the binding buffer and stained with FITC-Annexin V (Sigma-Aldrich) and PI solution for 30 min in the dark. Then, the sample was evaluated by the flow cytometer. A quadrant diagram of flow cytometry indicated the discrimination between living cells (Q4), early apoptotic cells (Q3), late apoptotic cells (Q2), and necrotic cells (Q1). The corresponding histograms of flow-cytometry showed the cellular apoptotic rates including early apoptotic cells (Q3) and late apoptotic cells (Q2).

### Western blot

Total protein was extracted from transfected cells using RIPA buffer supplemented with phosphatase inhibitors and protease inhibitors on ice for 30 min. The BCA method was utilized to determine the concentration of protein. Next, the protein was equally separated on 10% SDS-PAGE (sodium dodecyl sulfate-polyacrylamide gel electrophoresis) and then transferred to PVDF (polyvinylidene difluoride) membranes. After blocking with 5% skimmed milk for 2 h, the membranes were incubated with primary antibodies against CDK2, p21, Bax, Bcl-2, cleaved caspase-3, GAPDH, and SOS1 overnight at 4°C. Then, the membranes were washed with TBST (Tris-buffered saline and Tween 20) buffer, incubated with horseradish peroxidase (HRP)-conjugated secondary antibodies for 1 h at 25°C, and visualized using the ECL detection system (Pierce Biotechnology, USA). The semi-quantitative analysis of the protein expression was performed with Quantity One software (Bio-Rad, USA). GAPDH was used as an internal control.

### Luciferase reporter assay

The interaction between miR-217 and LINC01268 or SOS1 was detected by luciferase reporter assay. LINC01268 or SOS1 with putative binding sequences of wild-type (WT) miR-217 was amplified by qPCR and then cloned into the pmirGLO vector (Promega, USA) to obtain LINC01268-WT or SOS1-WT, respectively. The corresponding mutated sequences (MUT) of putative binding sequences were also cloned into the pmirGLO vector (Promega) to obtain LINC01268-MUT and SOS1-MUT. Cells were incubated for 24 h, and then co-transfected with LINC01268-MUT, SOS1-MUT, LINC01268-WT, or SOS1-WT and miR-217 mimic or control mimic using Lipofectamine 3000 reagent (Invitrogen). After 48 h of transfection, the dual-luciferase reporter assay system (Promega) was utilized to examine the luciferase activity, which was normalized to Renilla luciferase activity.

### RNA immunoprecipitation (RIP) assay

The binding relationship between miR-217 and LINC01268 was further validated by the RIP assay. Cells were lysed using RIP lysis buffer with a protease inhibitor cocktail and RNase inhibitor in a Magna RIP™ RNA-Binding Protein Immunoprecipitation Kit (Millipore, USA). The lysate was centrifuged at 20,000 *g* for 10 min at 4°C, and the supernatant was collected for immunoprecipitation. Then, the supernatant was mixed with RIP buffer containing a magnetic bead conjugated with human anti-Ago2 antibody or mouse immunoglobulin G (IgG, Millipore, USA). Next, beads were pelleted by centrifuging briefly at 10,000 *g* at 4°C, washed with RIPA buffer and PBS, and re-suspended with proteinase k. The mixture was centrifuged at 20,000 *g* for 10 min at room temperature, and the supernatant was divided into a 3:1 ratio for RNA and protein extraction.

### Statistical analysis

Data are reported as means±SD and were evaluated by SPSS 13.0. Each experiment was repeated at least three times. Student's *t*-test identified the significance of the difference between two groups, and one-way ANOVA analyzed the difference among multiple groups. P<0.05 was considered to be statistically significant. Survival curves were estimated by the Kaplan-Meier method followed by the log-rank test for the comparison of differences in survival distributions. A chi-squared test was conducted to evaluate the relationship between LINC01268 expression and clinical-pathological characteristics.

## Results

### LINC01268 expression was up-regulated in AML tissues and cells

**Figure 1:**
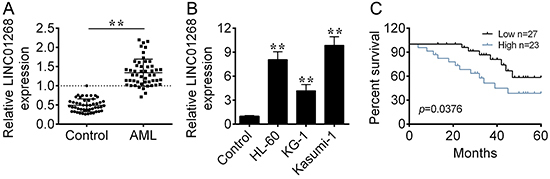
LINC01268 expression was up-regulated in acute myeloid leukemia (AML). **A**, qRT-PCR was applied to measure the expression of LINC01268 in bone marrow tissues from AML patients and healthy donors (control). **B**, The expression of LINC01268 in human AML cell lines (HL-60, KG-1, and Kasumi-1) and human bone marrow stromal cell line HS-5 (control) was determined by qRT-PCR. **C**, Survival curves of AML patients were plotted by the Kaplan-Meier method with the log-rank test. Data are reported as means±SD. **P<0.01 *vs* the control group (ANOVA and *t*-test).

The expression of LINC01268 was significantly upregulated by 1.6-fold in bone marrow samples from AML patients compared to that from healthy donors (Figure 1A). The expression of LINC01268 was also highly up-regulated in human AML cell lines (HL-60, KG-1, and Kasumi-1) compared to human bone marrow stromal cell line HS-5 (Figure 1B).

The expression level of LINC01268 was positively correlated with the number of white blood cells (P=0.001), hemoglobin level (P=0.007), and platelet count (P=0.001). There was no significant correlation between LINC01268 level and age, gender, FAB subtype, or complete remission (all P>0.05).

Based on the median expression value of LINC01268, AML patients were classified into two groups: LINC01268 high expression group (n=23) and LINC01268 low expression group (n=27). AML patients with high expression of LINC01268 had a worse overall survival compared to that with low expression of LINC01268 (Figure 1C).

### LINC01268 knockdown inhibited viability and promoted apoptosis of AML cells

**Figure 2:**
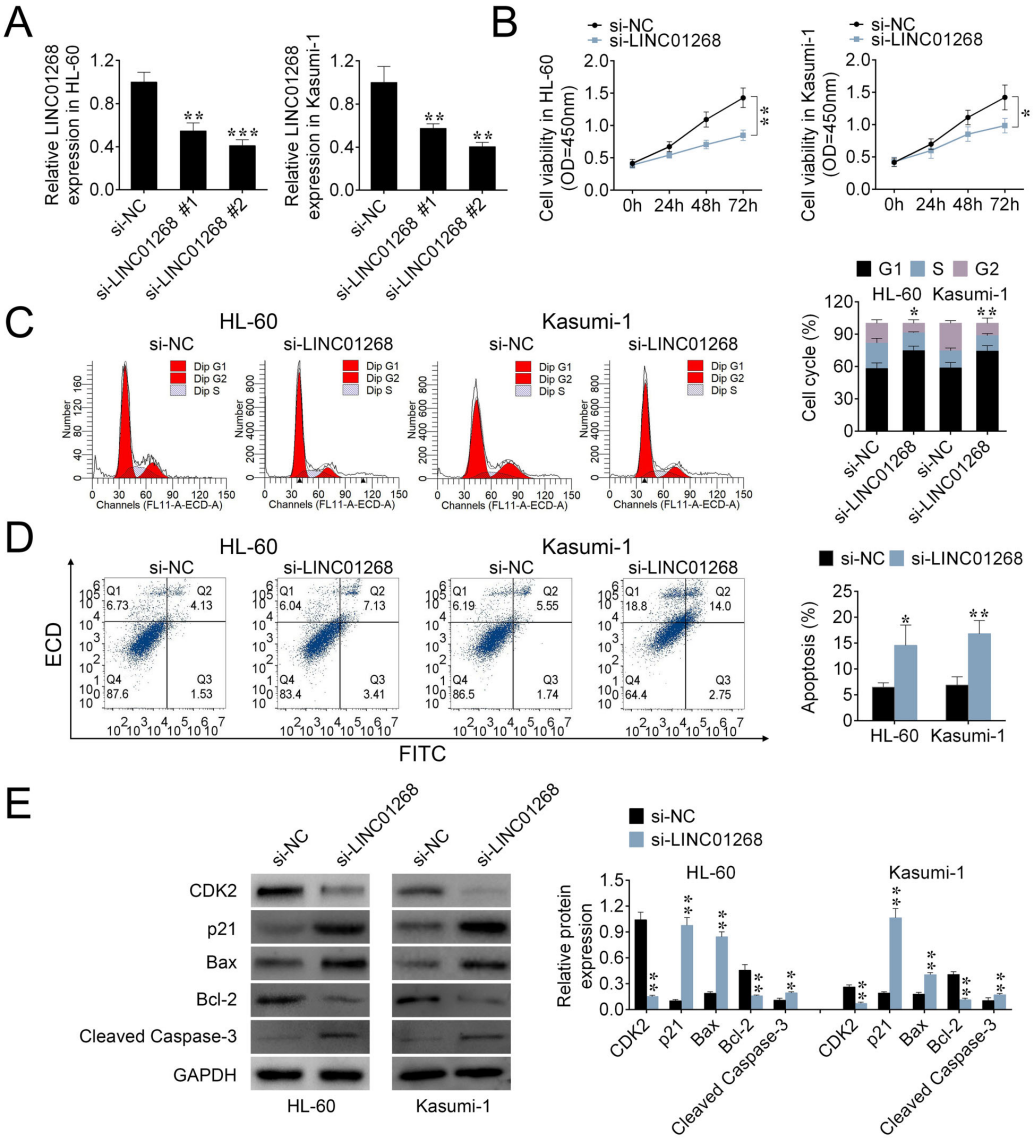
The effects of LINC01268 on acute myeloid leukemia (AML) cells. **A**, The expression of LINC01268 was detected by qRT-PCR in HL-60 and Kasumi-1 cells transfected with LINC01268 siRNAs (si-LINC01268 #1, si-LINC01268 #2). **B**, The viability of the transfected cells was examined by CCK-8. **C** and **D**, Cell cycle and apoptosis of the transfected cells were detected by flow cytometry. **E**, Protein expression levels of CDK2, p21, Bax, Bcl-2, and cleaved caspase-3 were determined by western blot. Data are reported as means±SD. *P<0.05, **P<0.01, ***P<0.001 *vs* the control group (NC) (ANOVA and *t*-test).

To explore the role of LINC01268 in AML cells, the cell viability, cell cycle stage, and cell apoptosis were determined in HL-60 and Kasumi-1 cells. The siRNAs for LINC01268 (si-LINC01268 #1, si-LINC01268 #2) were transfected into HL-60 and Kasumi-1 cells, and si-LINC01268 #2 possessed a lower expression level than si-LINC01268 #1, which was used in the following experiments (Figure 2A).

LINC01268 knockdown significantly inhibited the viability of AML cells (Figure 2B). For cycle stage analysis, LINC01268 knockdown increased the percentage of AML cells in the G1 phase, and decreased the percentage of cells in the G2 and S stages (Figure 2C). In addition, compared with the control group, LINC01268 knockdown caused a significant reduction in the protein expression of CDK2, and enhanced p21 protein expression (Figure 2E). CDK2 is a key regulator for cell division cycle (17) and p21 is regarded as a potent cyclin-dependent kinase inhibitor (18), which indicated that LINC01268 knockdown could regulate cell cycle progression.

LINC01268 knockdown significantly increased the apoptotic rates of AML cells compared to the control group (Figure 2D). Additionally, increased expression of pro-apoptotic proteins (cleaved caspase-3 and Bax) and decreased expression of an anti-apoptotic protein (Bcl-2) validated that LINC01268 knockdown promoted the apoptosis of AML cells.

### LINC01268 was identified as a sponge of miR-217

**Figure 3:**
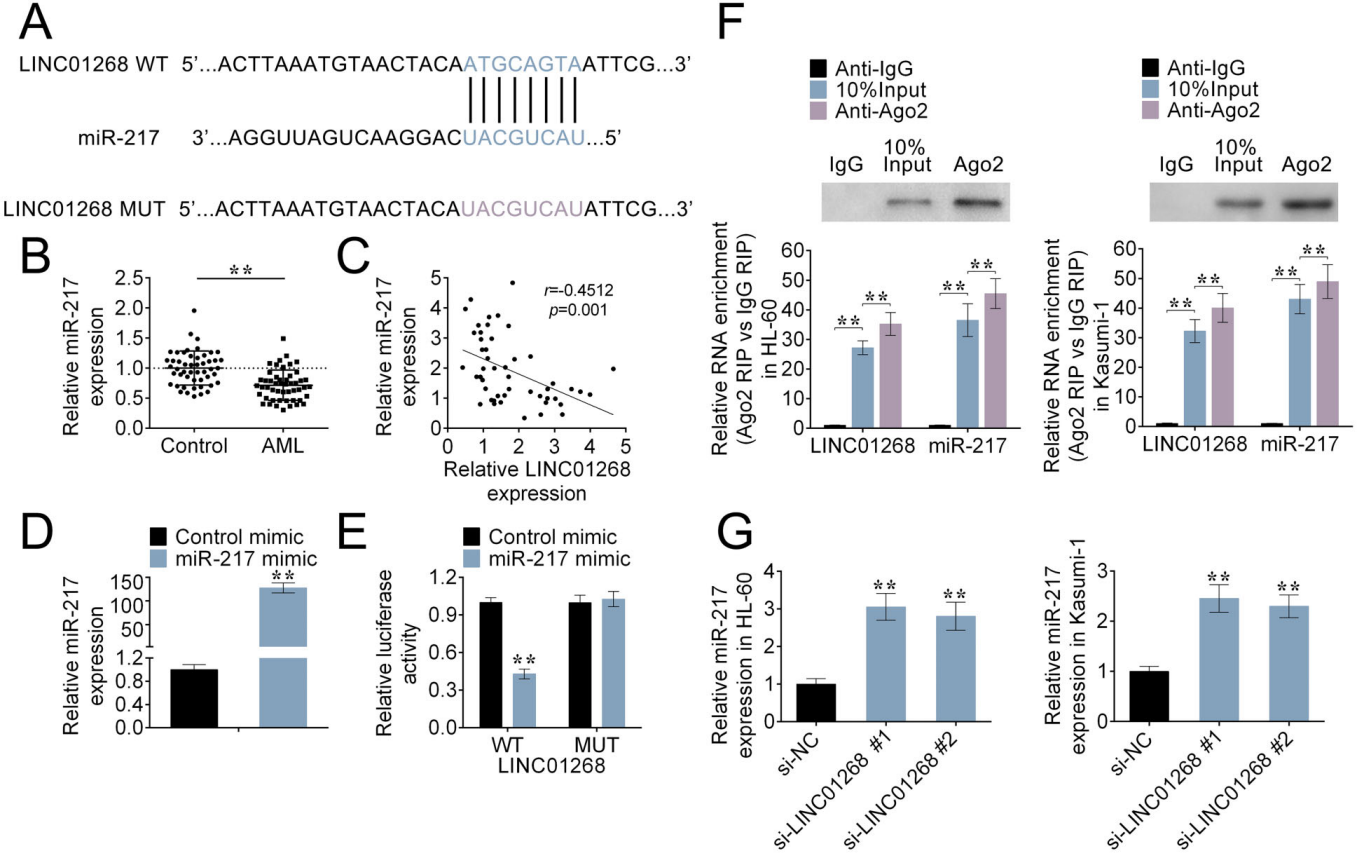
LINC01268 acting as a sponge of miR-217. **A**, The potential targeted miRNA for LINC01268 was identified by the miRDB website. **B**, The expression of miR-217 in bone marrow tissues from acute myeloid leukemia (AML) patients and healthy donors (control) was evaluated by qRT-PCR. **C**, The correlation between LINC01268 and miR-217 was evaluated by the Pearson correlation analysis. **D**, The expression of miR-217 in AML cells transfected with miR-217 mimic was detected by qRT-PCR. **E**, The luciferase activity of the reporter with LINC01268 wild type (WT) or mutated (MUT) was evaluated by the luciferase reporter assay. **F**, RIP assay demonstrated that miR-217 and LINC01268 were greatly enriched in the Ago2 antibody group. **G**, The binding relationship between miR-217 and LINC01268 was further confirmed by RIP assay. Data are reported as means±SD. **P<0.01 *vs* the control group (ANOVA and *t*-test). NC: control.

Through the miRDB website (http://www.mirdb.org/), miR-217 was predicted as a target of LINC01268 (Figure 3A). Interestingly, the expression of miR-217 in bone marrow tissues from AML patients was greatly down-regulated compared to that from the healthy donors (Figure 3B). Moreover, the Pearson correlation analysis showed a negative correlation between miR-217 expression and LINC01268 level (Figure 3C).

To verify the above findings, AML cells were transfected with miR-217 mimic or control mimic, the expression of miR-217 was markedly increased compared to the control group (Figure 3D). The luciferase activity of the reporter containing LINC01268 WT was down-regulated in AML cells co-transfected with miR-217 mimics, while the luciferase activity in the LINC01268 MUT reporter had no significant difference between miR-217 mimic and control groups (Figure 3E). Thus, LINC01268 could directly target miR-217. RIP assay further demonstrated that miR-217 and LINC01268 were greatly enriched in Ago2 antibody group compared to the control IgG antibody group (Figure 3F).

Additionally, qRT-PCR analysis showed that the expression of miR-217 in AML cells could be negatively regulated by LINC01268 (Figure 3G). Collectively, LINC01268 functioned as a ceRNA for miR-217.

### SOS1 was a target of miR-217

**Figure 4:**
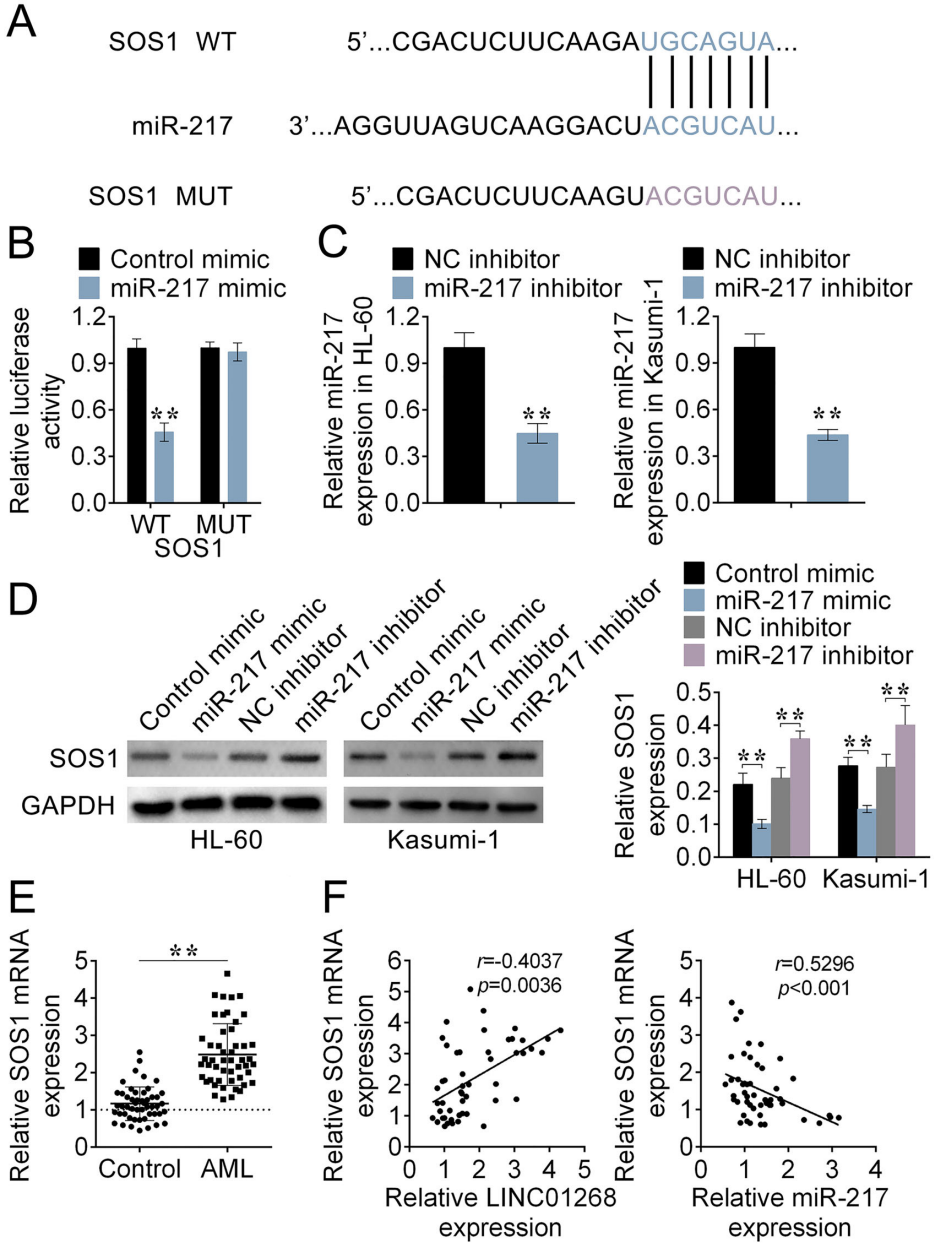
SOS1 was a target of miR-217. **A**, The relationship between miR-217 and SOS1 was predicted by TargetScan software. **B**, The luciferase activity of the reporter with SOS1 wild type (WT) or mutated (MUT) was evaluated by the luciferase reporter assay. **C**, After the transfection of miR-217 inhibitor, the expression of miR-217 in HL-60 and Kasumi-1 cells was measured by qRT-PCR. **D**, After acute myeloid leukemia (AML) cells were transfected with miR-217 mimic, control mimic, miR-217 inhibitor, or negative control (NC) inhibitor, the protein expression of SOS1 was determined by western blot. **E**, The expression of SOS1 in bone marrow tissues from AML patients and healthy donors was calculated by qRT-PCR. **F**, The correlation among SOS1, miR-217, and LINC01268 was evaluated by the Pearson correlation analysis. Data are reported as means±SD. **P<0.01 *vs* the control group (ANOVA and *t*-test).

TargetScan website (http://www.targetscan.org/vert_71/) predicted that SOS1 was a target of miR-217 (Figure 4A). To validate this, the luciferase activity of the SOS1 WT reporter was decreased after cells were co-transfected with miR-217 mimic compared to the control mimic group, and yet the activity in the SOS1 MUT reporter had no significant difference between two groups (Figure 4B). Thus, SOS1 was a direct target of miR-217.

The analysis of qRT-PCR showed the high transfection efficiency of miR-217 inhibitor in HL-60 and Kasumi-1 cells (Figure 4C). Protein expression of SOS1 in AML cells was decreased by transfection of miR-217 mimic, but increased by the transfection of miR-217 inhibitor (Figure 4D). Hence, miR-217 negatively regulated the expression of SOS1.

Furthermore, qRT-PCR verified that SOS1 expression was up-regulated in bone marrow tissues of AML patients compared to that of healthy donors (Figure 4E). The Pearson correlation analysis further suggested that SOS1 was negatively regulated by miR-217, and positively regulated by LINC01268 (Figure 4F).

### LINC01268 regulated viability, cycle progression, and apoptosis of AML cells through the miR-217/SOS1 axis

**Figure 5:**
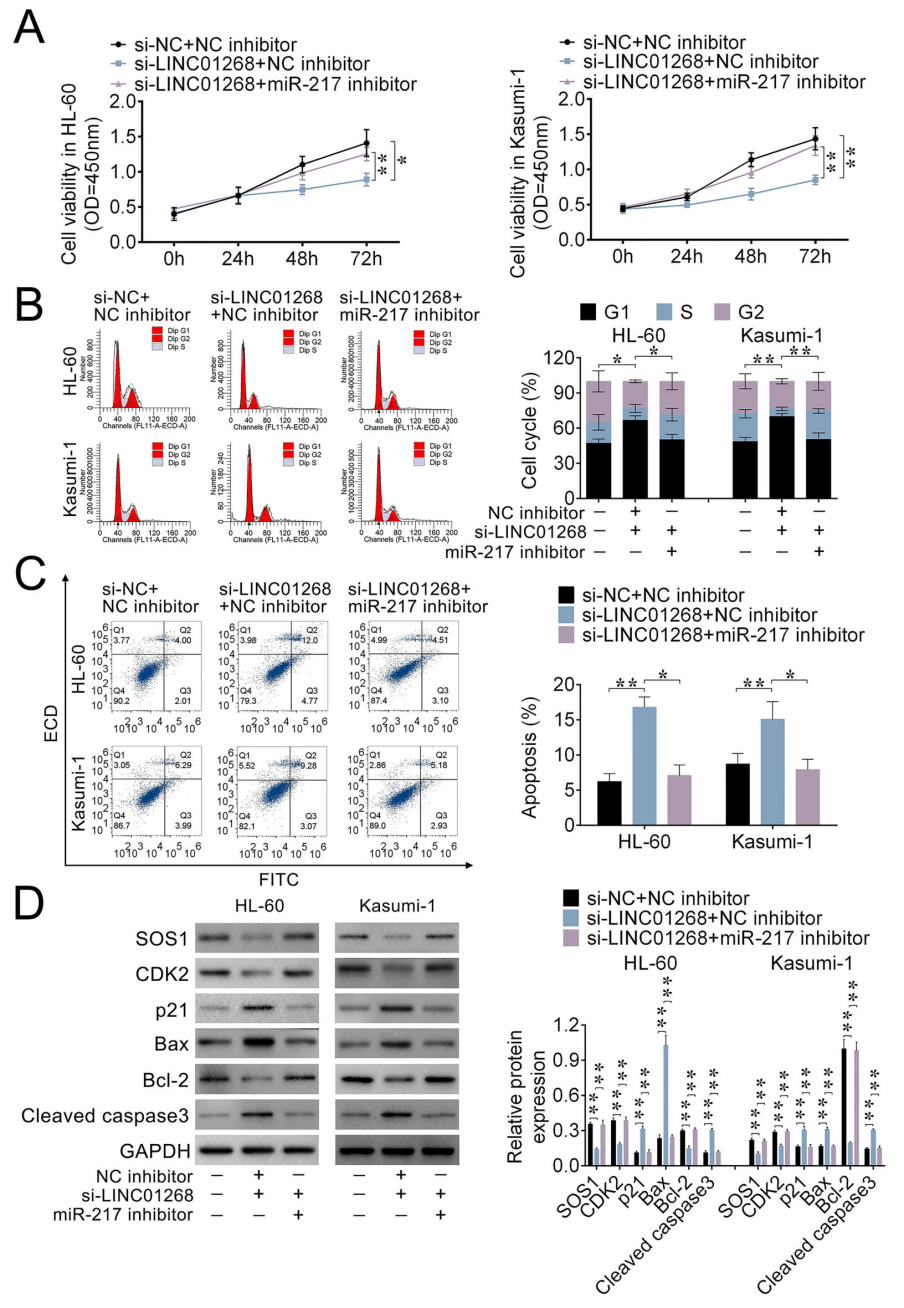
The roles of the LINC01268/miR-217/SOS1 axis in acute myeloid leukemia (AML) cells. After HL-60 and Kasumi-1 cells were transfected with si-LINC01268+miR-217 inhibitor, si-LINC01268+NC (negative control) inhibitor, or si-NC+ NC inhibitor, (**A**) the cell viability was detected by CCK-8 assay; (**B** and **C**) the cell cycle and apoptosis were measured by flow cytometry; and (**D**) the protein expression of SOS1, CDK4, p21, Bax, Bcl-2, and cleaved caspase-3 were evaluated by western blot. Data are reported as means±SD. **P<0.01 *vs* the control group (ANOVA and *t*-test).

The viability, cycle progression, and apoptosis of AML cells were determined to further explore the relationship among LINC01268, miR217, and SOS1 in HL-60 and Kasumi-1 cells. LINC01268 knockdown (si-LINC01268) inhibited the viability of AML cells, but enhanced the cell viability after co-transfection with si-LINC01268 and miR-217 inhibitor (Figure 5A).

For cycle progression analysis, compared with the control group, LINC01268 knockdown increased the percentage of AML cells in the G1 stage but decreased it in the G2 and S stages. This result was reversed after co-transfection with si-LINC01268 and miR-217 inhibitor (Figure 5B).

The apoptosis analysis suggested that the increased apoptotic rate of AML cells caused by LINC01268 knockdown was reversed after transfection of miR-217 inhibitor (Figure 5C).

Finally, Figure 5D indicated that the decreased SOS1 expression caused by LINC01268 knockdown was reversed by co-transfection of miR-217 inhibitor, which further confirmed the relationship among LINC01268, miR-217, and SOS1. LINC01268 knockdown increased the protein expression of p21, Bax, and cleaved caspase3, and decreased the protein levels of CDK2 and Bcl-2 (Figure 5D). However, these expressions were reversed by co-transfection of si-LINC01268 and miR-217 inhibitor. Therefore, LINC01268 promoted AML cell viability and cell cycle progression, and inhibited apoptosis through regulating the miR217/SOS1 axis.

## Discussion

Given the limited therapeutic methods and poor prognosis of AML patients, it is urgent to find new therapies for AML (5,6). lncRNAs have been reported to be critical for regulating gene expression, and their roles in the progress of AML have received much attention in recent years (8). LINC01268, a newly identified lncRNA, has been previously reported to play roles in the development of glioma, and its expression is methylation-dependent (14). Particularly, Lei et al. suggested that LINC01268 was associated with poor prognosis of AML patients (15). However, the roles of LINC01268 in AML remain unclear. The present study found that LINC01268 was highly expressed in AML patients compared with healthy donors. The over-expression of LINC01268 was associated with worse prognosis in AML patients. Additionally, the high expression of LINC01268 in AML cells promoted cell viability and cycle progression and inhibited apoptosis. Thus, LINC01268 could be used as a potential therapeutic target and prognostic marker for AML.

lncRNAs regulate cellular processes through diverse molecular mechanisms in various diseases (19); specifically, lncRNAs function as ceRNAs via competitively binding to microRNAs to regulate cellular functions (20). The present study found that LINC01268 could serve as a ceRNA for miR-217, which has been previously regarded as a tumor suppressor in the progression of osteosarcoma and ovarian cancer (21,22). Besides, miR-217 suppressed TGF-β1-induced proliferation and migration of airway smooth muscle cells through targeting ZEB1 (23). Importantly, serum miR-217 expression has been reported to be considerably downregulated in AML patients, which is relevant to aggressive clinical characteristics (24). Consistent with previous reports, our study showed that miR-217 was remarkably decreased in AML patients. Further analysis identified that SOS1 was a target of miR-217.

SOS1, a guanine nucleotide exchange factor, catalyzes the exchange of GDP for GTP and activates Ras (25). SOS1 could act as an oncogene and play an important role in cancers (25,26). You et al. (27) suggested that SOS1 was relevant to leukemogenesis. Moreover, SOS1 has been found to affect RAS/MAPK and PI3K/AKT pathways to regulate various cellular processes (28,29). In this study, SOS1 expression was up-regulated in AML patients. SOS1 had a negative correlation with miR-217 but a positive correlation with LINC01268. In addition, our study showed that LINC01268 regulated SOS1 expression to promote AML cell viability and cell cycle progression but inhibit cell apoptosis via sponging miR-217.

In conclusion, these results suggested that LINC01268 promoted AML cell viability and cell cycle progression but inhibited cell apoptosis by regulating miR-217/SOS1 axis. LINC01268 could be considered a potential biomarker for the therapy and diagnosis of AML. This study may offer a novel molecular mechanism for a better understanding on the pathology of AML. Certainly, this is a preliminary study considering the roles of LINC01268 in AML, and the promotion of the LINC01268/miR-217/SOS1 axis in the progression of AML still needs to be further validated by animal experiments and clinical trials.
